# Dental caries at enamel and dentine level among European adolescents – a systematic review and meta-analysis

**DOI:** 10.1186/s12903-022-02631-2

**Published:** 2022-12-18

**Authors:** Marit S. Skeie, Abhijit Sen, Göran Dahllöf, Tone Natland Fagerhaug, Hedda Høvik, Kristin S. Klock

**Affiliations:** 1 Department of Clinical Dentistry, Pediatric Dentistry, The Faculty of Medicine, University of Bergen, Årstadveien 19, 5009 Bergen, Norway; 2 Center for Oral Health Services and Research, Mid-Norway (TkMidt), Trondheim, Norway; 3 Department of Public Health and Nursing, Faculty of Medical and Health Sciences, Norwegian University of Science and Technology (NTNU), Trondheim, Norway; 4 Division of Orthodontics and Pediatric Dentistry, Department of Dental Medicine, Karolinska Institutet, Stockholm, Sweden

**Keywords:** Epidemiology, Caries prevalence, Caries experience, Adolescent

## Abstract

**Background:**

In contrast with the last century, caries epidemiology has begun integrating enamel caries into determinations of caries prevalence and experience. The objective of the present systematic review and meta-analysis was to assess the caries status including estimations of enamel caries, of European adolescents.

**Method:**

Four databases (Medline Ovid, Embase, CINAHL, and SweMed+) were systematically searched from 1 January 2000 through 20 September 2021 for peer-reviewed publications on caries prevalence and caries experience in 12–19-year-olds; that also included evaluations of enamel lesions. Summary estimates were calculated using random effect model.

**Results:**

Overall, 30 publications were selected for the systematic review covering 25 observational studies. Not all studies could be used in the meta-analyses. Caries prevalence was 77% (n = 22 studies). Highest prevalence was reported in the age groups 16–19 years, and in studies where caries examinations were done before 2010. The overall mean DMFT score was 5.93 (n = 14 studies) and it was significantly lower among Scandinavian adolescents than among other European adolescents (4.43 vs. 8.89). The proportion of enamel caries (n = 7 studies) was 50%, and highest in the lowest age group (12–15 years). Results from the present systematic review reflected the caries distribution to be skewed at individual-, tooth- and surface levels; at tooth and surface level, also changed according to age.

**Conclusions:**

Although studies in which the caries examinations had been done in 2010 or later documented a reduction in caries prevalence, caries during adolescence still constitutes a burden. Thus, the potential for preventing development of more severe caries lesions, as seen in the substantial volume of enamel caries during early adolescence, should be fully exploited. For this to happen, enamel caries should be a part of epidemiological reporting in national registers.

**Supplementary Information:**

The online version contains supplementary material available at 10.1186/s12903-022-02631-2.

## Introduction

Oral disease continues to constitute a global public health challenge. The most common oral disease globally is dental caries [1]. When occurring in the childhood years, caries may develop into a lifelong condition that tracks across adolescence and adulthood. Thus, it is worrying that in 2010, untreated caries in deciduous teeth was the tenth most prevalent health condition, affecting 9% of the global child population [1]. Surprisingly, from 1990 to 2015, global prevalence of untreated caries in deciduous and permanent teeth remained relatively unchanged [2]. These data also reveal that caries affected the permanent teeth of 5 billion people, with prevalence peaking in the 15–19-year-old group [2]. Although largely preventable, caries continues to be widespread, especially in many low- and middle-income countries [1]. In contrast, high-income countries have experienced a decrease in caries, most distinctly among 12-year-olds [3].

While a substantial number of epidemiologic studies have targeted childhood caries, few have focused on adolescents. Adolescence has been described as a period of continued behavioural development along a pathway established in childhood [4]. It is a critical life phase when the individual develops independence; peer interactions are gradually increasing, and parental control lessens. As a consequence, adolescent behaviour patterns differ from those in childhood and adulthood. Risk of caries in this phase of life is higher due to environmental factors such as a changing, sometimes poor, diet [5]; a lowering of oral hygiene standards [6, 7]; and a new independence for seeking, or avoiding, dental care [8]. The 12–15-year-old age group also faces a greater caries risk [9] due to newly erupted permanent canines, premolars, and second molars; 76 new tooth surfaces become exposed during this period. Adverse conditions around emerging teeth are another risk factor; good oral hygiene may be difficult, resulting in bacterial accumulation, which would promote the initiation of caries [10]. If favourable oral hygiene behaviours are not established before this period, it will be challenging for adolescents to maintain proper oral health hygiene [11].

Mejàre et al. observed a higher incidence of enamel caries on proximal surfaces among adolescents aged 12–15 years when compared to 20–27-year-olds [12]. The research group also found that the 12–15-year age group had a higher rate of caries lesion progression from the enamel-dentin border to the outer dentin compared with young adults [12]. In another study, Mejàre et al. [13] found that 11–12-year-old individuals with proximal caries experience showing visible radiolucency on bite-wing radiographs (BW) have a 2.5 times greater risk of developing new proximal enamel lesions than their counterparts with no such radiolucency. Caries when detected at the enamel stage, can be arrested or reversed, given the initiation of preventive strategies and non-operative treatment; thus, establishing good dental health habits, is clearly important.

Reproducible methods of dental caries evaluation have been described and measured for more than 70 years [14]. Even at that time, researchers were conscious of the possibility of caries arrest (inhibition of caries progression) and of the importance of an exact diagnosis of incipient or enamel caries as a therapeutic measure. Currently, inter-examiner reproducibility for enamel caries is acceptable, mostly due to the development of scientifically proven caries diagnostic criteria and examiner calibration routines [15, 16]. Regretfully, today national epidemiological surveys rarely assess enamel caries [17]. Caries prevalence in the population is thus underestimated, and the usefulness of the survey data in oral health care planning is undermined. However, a growing awareness is seen of the predictive strength of enamel lesions and their role in risk assessment [18], also, in the potential for managing future caries development through early, non-invasive treatment [19, 20]. Additionally, reporting caries patterns with enamel caries included at the individual, tooth, and surface levels is recognized as important for planning and evaluating oral health care [21]. In a lifespan perspective, preventive and early non-invasive treatment in adolescents is essential; caries control in this period will lay the foundation for good oral health in adulthood and reduce future costs for restoration and repair [22].

Previous systematic review and meta-analyses on caries prevalence [2, 23, 24] have used the World Health Organization (WHO) caries diagnostic criteria [25] based on the Decayed, Missing, and Filled Teeth index (DMFT) [26]. By this criterion cavitation in the dentine is used for caries detection, thus ignoring the presence of enamel caries. Kale et al. [24] targeted children and adolescents aged 6–15 years in the Eastern Mediterranean region, while Kassebaum et al. [2] took a global perspective and included all ages. No systematic reviews and meta-analyses on caries have included a focus on enamel caries in a study population of European adolescents.

The aims of the present systematic review and meta-analyses were to determine the prevalence and experience of dental caries in European adolescents with particular emphasis on the role of enamel caries. Three research questions were investigated a) What is the overall caries prevalence and caries experience at various ages during adolescence, and do they vary by age, year of publication, year of caries examination, type of caries examination or geographical region? b) What proportion of the total caries experience does enamel caries constitute at various ages? c) What is the caries distribution at various ages during adolescence at the individual-, tooth-, and surface levels?

## Methods

### Search methods

Four electronic databases (Medline Ovid, Embase, CINAHL, and SweMed+) were systematically searched from 1 January 2000 through 20 September 2021. We also manually searched the reference lists of all included publications for other relevant citations. The search was restricted to publications published in peer-reviewed journals and written in English, German, Norwegian, Swedish or Danish. Additional file 1: 1 presents the search terms used in the four databases.

### Selection criteria

Reviews assessing prevalence data must adhere to the **C**o**C**o**P**op (**C**ondition, **C**ontext, and **P**opulation) mnemonic criteria [27]. The observational studies including cross-sectional, case–control, cohort designs (prospective or retrospective), and randomized controlled trials (RCTs) (*e.g.*, caries baseline reports before intervention, or caries data from the control group) were included.

#### *P*opulation

Adolescents 12–19 years living in Europe were selected to limit the populations to a more comparable Human Development Index (HDI) country (https://en.wikipedia.org/wiki/List_of_sovereign_states_in_Europe_by_Human_Development_Index) than if the same age group of the global population was selected. Table 1 outlines the characteristics of the studies and participants: publication year; year of examination; country; levels according to national, subnational (regions), and community (cities and small areas); gender; socio-economic status or position (SES/SEP); immigrant background; and age.

**Table 1:** Background characteristics, applied examination and assessment and the targeted primary outcomes

Background characteristics						Examination and Assessment			Primary outcomes			
First Author, Publication year	Year of exam Country Level	Study design	Sample size (N) Total F: Female M: Male	Socio-economic status/ -position (SES/SEP) Immigrant (IM) + : reported − : not reported	Age (yrs)	Diagnostic method (ref.)	BW (Yes/No)	Calibration (Yes/No) Examiners (N)	Caries prevalence (%) (D(M)FS > 0 or D(M)FT > 0) 1: enamel level 2: dentine level	Caries experience Mean D_e_S Mean D(M)FS or Mean D_e_T Mean D(M)FT with enamel caries included	Proportion (D_e_S/D(M)FS or Proportion (D_e_T/D(M)FT	Caries distribution + : reported −: not reported
*Full mouth caries examination*												
Saethre-Sundli HB et al. [49] 2020	2014 Norway Subnational	Cohort Exam. at Follow-up 2014	3,282 *F: 1586 M: 1696*	+ : Parental education Family status National background	Mean age: 12.1 (SD: 0.5)	Amarante E et al. [71]	Yes	Yes 91	1: 58 2: 32	D_e_S: 1.35 DMFS: 2.15 (SD: 3.4)	0.63	+
Jacobsen ID et al. [43] 2016	2010–2011 Norway Subnational	Cross-sectional	869 F: 420 M: 449	+ : National background Parental education Family status	16	Amarante et al. [71]	Yes	Yes 1	1: 94 2: 83	-	-	-
David J et al. [59] 2006	1993 Baseline	Cohort Exam. at Baseline	159	+ : Mothers’ education	12	Amarante E et al. [71]	Yes	Yes 5	1: 90 2: 63	D_e_S: 6.2 (SD: 5.9) DMFS: 8.9 (SD: 7.8)	0.70	+
	1999 Follow-up Norway Community	Exam. at Follow-up	112 F: 56 M: 56		18	Amarante E et al. [71]	Yes	Yes 1	1: 99 2: 92	D_e_S: 4.4 (SD: 4.5) DMFS: 13.1 (SD: 11.4)	0.34	
Karlsson F et al [51] 2019	2009 Baseline	Cohort Exam. at Baseline	159 F: 82 M: 77	-	12	Socialstyrelsen, 1988 [72]	BW on indication only	No -: number examiners not reported	1: 48 2: not reported	*D_e_S: 1.1 (SD: 2.3) DFS: 1.8 (SD: 2.9)	*0.61	-
	2014 Follow-up Sweden Community	Exam. at Follow-up	159 F: 82 M: 77		17				1: 55 2: not reported	*D_e_S: 2.6 (SD: 4.2) DFS: 4.5 (SD: 6.1)	*0.58	
Koch G et al. [54] 2017	2013 Sweden Community	Cross-sectional	101 F: 49 M: 52	-	15	Koch G [73]	Yes	Yes 3	1: 57 2: not reported	DFS: 3.0 (CI: 1.9–4.1)	-	+
Jacobssen B et al. [74] 2011	2003 Sweden Community	Cross-sectional	85 IM: 11	+ : Education National background	15	Koch G [73]	Yes	Yes 8	1: 81 2: not reported IM: 1: 100 2: not reported	- IM DFS: 11.8 (CI: 5.4–18.3)	-	-
			Non IM: 74						Non IM: 1: 78 2: not reported	Non IM: DFS: 5.5 (CI: 3.9–7.1)		
Hugoson A al. [55] 2008	2003 Sweden Community	Cross-sectional	96 F: 51 M: 45	-	15	Koch G [73]	Yes	Yes 10	1: 80 2: not reported	D_e_S: 4.7 DFS: 6.4 (CI: 4.8–8.0)	0.73	+
Agustsdottir H et al. [50] 2010	2004–2005 Iceland National	Cross-sectional	757	-	12	ICDAS	Yes	Yes 1	1: 85 2: 66	*D_e_S: 5.67 (SE:0.47) DMFS: 8.77 (SE:0.64)	*0.65	+
			750		15				1: 94 2: 80	*D_e_S: 10.66 (SE: 0.80) ( DMFS: 17.00 (SE:1.10)	*0.63	
Splieth CH et al.[45] 2019	2016 Germany National	Cross-sectional	55,002	+ : School type Class level	12	WHO [25] + initial caries lesions (IT)	No	Yes 482	1: 34 2: 21	D_e_**T**: 0.52 DMF**T**: 0.96	0.54	+
Jablonski-Momeni A et al. [46] 2014	2009–2010 Germany Subnational	Cross-sectional	2 regions (969) Region 1: 525	+ : Mothers’ education National background	12	ICDAS	No	Yes	1: 43 2: 23	*D_e_S: 0.77 DFS: 1.61	*0.48	+
			Region 2: 444					Yes 1	1: 50 2: 17	*D_e_S: 1.7 DFS: 2.80	*0.61	
Wang X et al. [37] 2021	2013 England, Wales, Northern Ireland National	Cross-sectional(analyses of clusters)	2160 Cluster analyses:	+ : School type Free school meals eligibility Deprivation Index (IMD) Region category National background	15	ICDAS [75]	No	From dental records (available data)	1: not reported 2: not reported	D_e_S: 1.73 DMFS: 4.39 CI: 3.60- 5.18)	0.39	+
Wang X et al. [21] 2021	2013 England, Wales, Northern Ireland National	Cross-sectional	2532	+ : School type Free school meal eligibility Deprivation Index (IMD) Region category National background	12	ICDAS	No	Yes 75	1: 65 2: 45	D_e_S: 1.61 DMFS: 3.91 (SD: 5.90)	0.41	+
			2418		15				1: 73 2: 59	D_e_S: 2.02 DMFS: 5.94 (SD: 8.04)	0.34	
Vernazza CR et al. [38] 2016	2013 England, Wales, Norhern Ireland National	Cross-sectional	9866 (number included also 5-, 8- year-olds)	+ : Free school meals eligibility Gender	12	ICDAS	No	Yes 75	1: 57 2: approximately 33	D_e_**T**: 1.2 DMF**T**: 2.0	0.60	-
					15				1: 63 2: approximately 50	D_e_**T**: 1.5 DMF**T**: 2.9	0.52	
Baciu D et al. [52] 2015	2011 UK Community (n = 5)	Cross-sectional	592 F: 323 M: 269	+ : Regions	Mean age: 12.3	ICDAS	No	Yes 1	1: 83 2:76	*D_e_S: 1.71 (SD: 2.10) DMFS: 6.78 (SD: 7.25)	*0.25	-
Maldupa I et al. [48] 2021	2016 Latvia National	Cross-sectional	2138 F: 1031 M: 1107	+ : Region Socio-economy (Family Affluence Scale (FAS))	12	ICDAS II	No	Yes 7	1: 99 2: 80	*D_e_S: 12.6 (SD: 10.5) DMFS: 17.6 (SD: 13.2)	*0.72	+
Deery C et al. [76] 2000	1997 Latvia Community	Cross-sectional	182 F: 102 M: 80	-	Mean age: 13.3 (range: 10.6–15.7)	Deery et al. [77]	No Visual examination (CVE)	Yes 1	1: not reported 2: 99.5	D_e_S: 10.38 (SD: 11.68) DMFS: 22.65	0.46	-
Almerich-Torres T et al. [41] 2020	2018 Spain Community	Cross-sectional	632	+ : Social class by parental occupation	12	ICDAS II	No	Yes 3	1: not reported 2: 30	D_e_S: 1.67 DMFS: 2.41	0.69	-
			534		15				1: not reported 2: 45	D_e_S: 2.03 DMFS: 3.39	0.60	
Almerich-Torres T et al. [39] 2017	2010 Spain Community	Cross-sectional	409 F: 216 M: 193	+ : Social class by parental occupation (Domingo et al.)	12	ICDAS II	No	Yes 3	1: not reported 2: not reported	D_e_**T**: 2.57 DMF**T**: 3.44 (CI: 3.08–3.80)	0.75	-
			433 F: 226 M: 207		15					D_e_**T:** 3.66 DMF**T**: 4.74 (CI: 4.37–5.11)	0.77	
Almerich-Silla JM et al. [40] 2014	2010 Spain Community	Cross-sectional	1373	+ : Social class by parental occupation (Domingo et al.)	12	ICDAS II	No	Yes 6	1: 77 2: 38	D_e_S: 3.18 DMFS: 4.45 (CI: 3.96–4.93)	0.71	-
					15				1: 85 2: 44	D_e_S: 4.23 DMFS: 5.87 (CI: 5.36–6.37)	0.72	
Calado R et al. [47] 2017	2009 Portugal National	Cross-sectional	1309	+ : Mothers’ occupation and education Area of residence Region	12	ICDAS	No	Yes 24	1: 76 2: 47	D_e_S: 3.40 (SD: 0.17) DMFS: 8.61 (SD: 0.34)	0.39	+
			1075		18		No		1: 89 2: 68	D_e_S: 4.36 (SD: 0.20) DMFS: 16.64 (SD: 0.51)	0.26	
Campus G et al. [60] 2020	2017 Italy National	Cross-sectional	7064 F: 3605 M: 3459	+ : Income inequality and Unemployment rate Parental education Working status National background	12	ICDAS [78]	No	Yes 4	1: 70 2: not reported	D_e_**T**: 2.29 DF**T**: 3.71	0.62	-
Diamanti I et al. [57] 2021	2013 Greece National	Cross-sectional	1243 F: 631 M: 612	+ : Urban/rural area of residence Parental education	12	ICDAS II	No	Yes 10	1: 72 2: 52 1: F: 74 1: M: 69 2: F: 54 2: M: 50	- *F: D_e_**T:** 1.8 (SD: 2.5) DMF**T**: 3.6 *M: D_e_**T**: 1.7 (SD: 2.6) DMF**T**: 3.1	*F: 0.50 *M: 0.55	-
			1227 F: 658 M: 569		15				1: 82 2: 66 1: F: 79 1: M:86 2: F: 68 2: M: 63	- *F: D_e_**T**: 2.6 (SD: 3.2) DMF**T**: 5.2 *M: D_e_**T**: 2.4 (SD: 3.1) DMF**T**: 4.7	*F: 0.50 *M: 0.51	
***Partial mouth caries examination (proximal surfaces of posterior teeth). One study also included occlusal surfaces***												
Jacobsen ID et al 2019 [42]	Jacobsen ID et al. Norway. Details under full mouth caries examination								1: 84 2: not reported	D_eA_S: 5.8 (SD: 5.0)	-	-
Bergström EK et al [53] 2019	2005-	Cohort Controls: Exam. at Baseline	Control group: 10,160 F: 4923 M: 5237	+ : Geographical area	12	Gröndahl et al. [80]	Not reported	Not reported	1: not reported 2: not reported	D_eA_S: 0.86 D_A_FS: 1.08	0.80	-
	2008 Sweden Subnational	Controls: Exam. at Follow-up			15					D_eA_S:2.19 D_A_FS: 2.88	0.76	
Alm A et al. [56] 2006	2002 Sweden Community	Cohort Exam. at Follow-up	568 F: 286 M: 282 ???	+ : Socio-economic regions	15	Socialstyrelsen [72]	Yes	Yes 1	1: 67 2: 22	D_eA_S: 2.78 (range 0–18) D_A_FS: 3.23 (Range:0–20)	0.86	-
**Koch G et al [54] 2017	2013 Jönköping study, Sweden. Details under full mouth caries examination										0.80–0.90	
Hugoson A et al. [55] 2008	2003 Jönköping study, Sweden. Details under full mouth caries examination										0.80–0.90	
Sköld UM et al. [79] 2005	1999–2003 Sweden Community	Cohort Controls: Exam. at Baseline	94	-	13	Gröndahl et al. [80]	Yes	No 2	1: not reported 2: not reported	D_A_FS: 1.45 (SD: 2.17)	-	-
		Controls: Exam. at Follow up			16				1: not reported 2: not reported	D_A._FS: 3.29 (SD: 4.45)		
Jacobsson B et al. [81] 2005	2003 Sweden Community	Cross-sectional	117 IM: 51 F: 27 M: 24	+ National background	15	Koch [73]	Yes	No 4	1: 74 2: 44 1: IM: 76 2: IM: 47	IM: D_eA_S: 5.5 (CI: 4.0–7.0) D_A._FS: 6.5 (CI: 4.7–8.2)	0.85	-
			Non IM: 66 F: 40 M: 26						1: Non IM: 74 2: Non IM: 42	Non IM: D_eA_S: 3.3 (CI: 2.3–4.4) D_A._FS:: 4.0 (CI: 2.9–5.4)	0.83	
Lith A et al. [82] 2002	1992–1993 Sweden Community	Cross-sectional data From dental records	285	+ : Income and education exceeded the average Swedish citizen	17	Also *occlusal lesions* Gröndahl et al. [80]	BW on indication only	Yes -: number examiners not reported	1: 93 2: 84	-	-	+
Gustafsson A et al. [83] 2000	1993	Cohort Exam. at Baseline	93 Analyzed 67 F: 34 M: 33	-	14	Gustafson et al. [83]	Yes	Yes 2	1: 69 1: F: 76 1: M: 62 2: not reported	- F: D_eA_S: 4.2 (SD: 5.5) D_A._FS: 4.82 M: D_eA_S: 2.9 (SD: 43.9) D_Al_FS: 3.62	- F: 0.87 M: 0.80	-
	1998 Sweden Community	Exam. at Follow-up	Analyzed 66 F: 33 M: 33		19				1: 92 1: F. 91 1: M: 94 2: not reported	- F: D_eA_S: 7.0 (SD: 4.7) D_A._FS: 9.7 M: D_eA_S: 6.1 (SD: 4.7) D_A_FS: 10.6	- F: 0.72 M: 0.58	
Pooerterman JHG et al [84] 2003	1990	Cohort From radiographs	121	-	14	Poorterman JHG. [85]	Yes	Yes 2	1: 86 2: not reported	D_eA_S:2.7 (SD: 3.1) D_A._FS: 3.73	0.72	-
	1993 The Netherlands Community	From radiographs	311		17				1: 88 2: not reported	D_eA_S: 3.8 (SD: 1.1) D_A._FS: 7.0	0.54	

The first part of the table consists of publications based on full mouth caries examination and the second one, on articles from partial caries examination (both places arranged after country of origin). Within these sections, the publications are presented consecutively according to publication date

D_e_S: Surfaces with enamel caries (without– and with cavitation). D_eA_S: Proximal (approximal) D_e_S. D_e_T: Teeth with enamel caries (without– and with cavitation). D(M)FS: decayed (**enamel and dentine caries**)/(missed)/filled permanent surfaces. D(M)FT: decayed (**enamel and dentine caries**)/(missed)/filled permanent teeth. In many studies the M-component is not counted because no or almost no teeth were extracted due to caries. If available surface level index, this the one which is denoted in the table. D_A_(M)FS: decayed (**enamel and dentine caries**)/(missed)/filled **approximal** surfaces. Measures of variability (e.g. SD) are lacking in those cases components of mean caries experience have been calculated. Dental caries activity was not assessed

(*) In case that enamel caries omits cavitated lesions in enamel and as such underscores the D_e_S/ D_e_T component

(**) Article denoted in both categories; full mouth- and partial caries examination

#### Condition

The selected studies reported on dental caries in permanent teeth. All of them incorporated enamel caries (enamel caries with and without cavitation) which clinically implied any sign of caries in the enamel, and when radiographs were used, any radiolucency in enamel*.* The examinations were carried out either by full-mouth or partial-mouth examination (examination of proximal lesions in posterior teeth). The outcome variables were caries prevalence at enamel threshold (D[M]FS [S: Surface] > 0 or D[M)]T > 0), prevalence at dentine threshold without enamel caries, mean total caries experience (mean D[M]FS or mean D[M]FT, including enamel lesions), the enamel caries proportion of this latter value, and presentations of caries distribution at the individual, tooth, and surface levels (Table 1).

#### Context

The context or specific settings relevant to caries prevalence and caries experience, were reported. The following subgroups were used in the meta-analyses: age (12–15 years vs. 16–19 years as well as 12–13 years vs. 16–19 years), publication year (< 2010 vs. ≥ 2010), caries examination year (< 2010 vs. ≥ 2010), mouth examination (full- vs. partial-mouth), and region (Scandinavia [Norway, Sweden, Denmark] vs. rest of Europe).

### Exclusion criteria

**Fig. 1:**
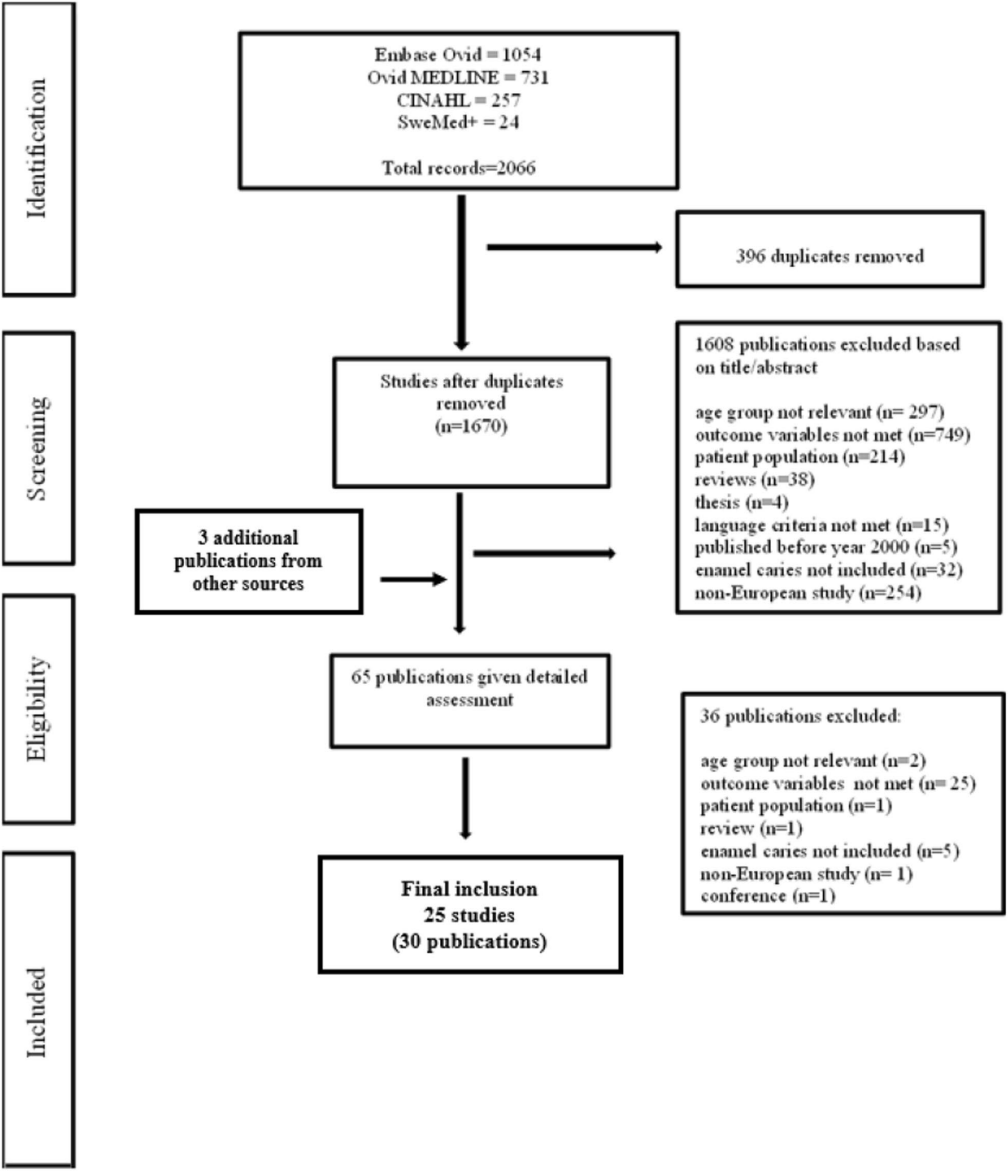
Flow chart

Figure 1 illustrates the selection of studies, with reasons for exclusion, in a flow chart. Studies not reporting enamel caries and studies examining groups with various medical problems were excluded. Studies comparing populations exposed to low or high-water levels of fluoride were also excluded [28–30]. Adolescents under 12 years of age were excluded to avoid results from the deciduous dentition being included in the data. Not all publications selected for the present systematic review could be included in the meta-analyses because some publications represented the same study and were considered as one study in the meta-analysis; some did not report the exact sample size of the adolescent groups, only the total sample size; and some only reported estimates of caries prevalence, not caries experience, or vice versa. Additionally, mean caries experience of enamel caries (mean D_e_S) or of total caries (mean D[M]FS with enamel lesions included), was sometimes reported without 95% confidence intervals (CI), standard deviations (SD) or Standard errors (SE). These studies were also excluded. Lastly, publications reporting total caries experience on the tooth level (D[M]FT) were also omitted as only a few did so and a meta-analysis can only be done on comparable values.

### Data extraction

Two reviewers (MSS, KSK) independently evaluated articles for inclusion in the study. Articles were first selected based on the title. The reviewers then read the abstracts of these articles, followed by the full-text article if the study was within the scope of the research questions in the present study. Both reviewers then re-read the full-text articles that had been selected to determine final inclusion in the study; in cases of doubt, a third author (AS) read the article and discussed it with the reviewers to reach a consensus.

### Critical appraisal

We assessed risk of bias using the Joanna Briggs Institute (JBI) Critical Appraisal Instrument for Studies Reporting Prevalence Data, a revision of the JBI critical checklists for studies reporting prevalence data [27, 31]. The instrument evaluates nine items. The quality assessment of studies included were performed by two authors (MSS and KSK). In case of discrepancies, a third author (AS) was consulted (Additional file 1: 2). The instrument’s range of scores was from 0 to 11. Based on the scoring, overall, the studies were of good scientific quality. Two studies were scored equal to 8, all the others above 8.

### Statistical analysis

The statistical analyses were conducted using Stata version 17.1 (StataCorp, College Station, TX, USA). *Metaprop,* a new command in Stata was used to conduct meta-analyses of proportions which allows computation of exact binomial confidence intervals using the ci (method) option [32]. The subgroups and overall summary estimates of dental caries prevalence with inverse-variance weights were obtained using random-effects model. The *metan* command was used to estimate overall caries experience and approximate proportion of enamel caries via pooling of study-specific estimates (mean enamel caries experience divided by mean total caries experience) and corresponding 95% confidence intervals, using inverse variance method of the Der Simonian and Laird random effect model. *Cochran’s Q test and I*^2^ [33] were used to assess heterogeneity between studies; I^2^ is the total variation explained by between-study variation. A value above 60% was considered to be substantial heterogeneity. The influence analyses were performed by removing one study at a time to assess whether a single study changed pooled estimates. Subgroup analyses were done to investigate potential sources of heterogeneity (studies within and between the groups). Conventional funnel plots for assessing the publication bias were found to be inaccurate to determine proportional related studies (i.e., for caries prevalence) [34], thus, LFK index to detect and quantify asymmetry of study effects in Doi plots. However, for mean caries experience, conventional Egger’s test [35] and Begg’s test [36] as well as funnel plots were inspected to assess publication bias. If *p* < 0.10 or if there was asymmetry in the funnel plots, the results were considered to indicate publication bias**.** Due to the low number of studies, no publication bias assessment was done for the meta-analysis of the proportion of enamel caries. Sensitivity analyses were done by omitting one study at a time to check the robustness of the findings. For each study, the displayed effect size corresponds to an overall effect size computed from a meta-analysis excluding that study. In addition, the plot also displays a vertical line at the overall effect size based on the complete set of studies (with no omission) to help detect influential studies.

## Results

In total, 30 publications (Table 1), all published in English, met the inclusion criteria for the present systematic review; together, these publications reported data on approximately 92,780 adolescents (the exact number is unknown since some samples included younger age groups). Europe currently (year 2021) comprises 44 countries (https://www.worldometers.info/geography/how-many-countries-in-europe/); these publications cover 11 of the countries (25%). No publication studied populations in the 14 European countries with the lowest economic background according to GDP per capita (Gross domestic product divided by the total population) (https://www.thetealmango.com/featured/poorest-countries-in-europe/).

Three publications used data from UK’s the 2013 Children’s Dental Health Survey (CDHS) [21, 37, 38]. For meta-analysis, we used the publication with the highest sample size [21]. Three publications used survey data from the Valencia region of Spain [39–41], again the publication with the highest sample size was included in the meta-analysis [40]. Finally, of the two publications from the oral section of the “Fit Futures” study in Troms county, Norway [42, 43], the study presenting full-mouth caries data was used for meta-analysis [43], because the other publication only partially covered the study. Hence, in total 25 studies were included (30 publications). The types of caries examination varied. Of 30 publications, 22 publications reported caries based on full-mouth examination, of these, two included both full and partial mouth data. Eight of the publications were solely based on partial mouth examinations.

Ten publications were from the 2000s, 14 from the 2010s and six from the 2020s. Swedish publications were in the majority (n = 11). The majority of all publications (n = 17) included caries data of 12-year-olds, either as the only age group or together with other age groups. All studies (n = 25) were observational, mostly with cross-sectional designs (n = 18). In studies with cohort designs (n = 7), examination data were collected cross-sectionally, at baseline, at follow-up, or at both sessions. Cohort studies with intervention collected caries data from the control group (n = 2). The International Caries Detection and Assessment System (ICDAS) [44] was the caries diagnostic method most often used, but its Code 3 (visual change in enamel with cavitation) could not be separately quantified in all studies and hence, was not included in the total magnitude calculated for enamel caries. Table 2 shows the criteria of the different diagnostic tools for enamel caries.

**Table 2:** The criteria for enamel caries (with and without cavitation) in the different diagnostic tools used

Diagnostic tools	Clinical examination	Radiographical examination
**Full mouth caries examination**		
**Amarante et al. 1998 **[71]		
*Grade 1*	Occlusal: White or brown discoloration in enamel. No clinical cavitation. No radiographic evidence of caries	Proximal: Radiolucency in outer half of enamel
*Grade 2*	Occlusal: Small cavity formation, or discoloration of the fissure with surrounding grey/opaque enamel and/or radiolucency in enamel on radiograph	Proximal: Radiolucency in inner half of enamel
***Socialstyrelsen, 1988 (National Board of Health and Welfare) ***[72]		
Initial caries (D_**i**_)	Loss of mineral in the enamel causing a chalky appearance but without any clinical cavitations	Not reported as radiographs were performed on individual indication only
***Koch G, 1967 ***[73]		
Initial caries	Loss of mineral in the enamel causing a chalky appearance but not clinically classified as a cavity	The lesion restricted to the enamel
***The International Caries Detection and Assessment System (many researchers have contributed developing the criteria ***[44, 75, 78]		
ICDAS (see https://www.ncbi.nlm.nih.gov/pmc/articles/PMC5030492/) ICDAS II criteria (see https://www.ncbi.nlm.nih.gov/pmc/articles/PMC5573507/)		
*Code 1*	Smooth tooth surfaces: A loss of mineral in the enamel causing a chalky appearance, but without any clinical cavitations	
*Code 2*	Distinct visual change in enamel: The tooth must be viewed wet. When wet there is a (i) carious opacity (white spot lesion) and/or (ii) brown carious discoloration which is wider than the natural fissure/fossa that is not consistent with the clinical appearance of sound enamel (Note: the lesion must still be visible when dry)	
*Code 3*	Localized enamel breakdown because of caries with no visible dentin or underlying shadow	
***Splieth CH *et al*., 2019 ***[45]		
IT Initial caries lesions with no precise description		
***Deery C *et al*., 1995 ***[77] **Clinical visuals examination (CVE) alone**		
W		
B	White spot enamel caries	
Brown spot enamel caries		
E	Enamel caries with breakdown of surface	
***Partial mouth caries examination (proximal surfaces of posterior teeth)***		
***Gröndahl *et al*., 1977 ***[80]		
Based on BW:		(1) Caries lesion in the outer half of the enamel
Based on BW:		(2) Caries lesion more than halfway through the enamel but not passing the enamel-dentin junction
**Poorterman HJ et al., 2003 **[84] Based on BW:		A lesion confined to the enamel
***Gustavsson *et al*., 2000 ***[83]		
Based on BW:		A lesion confined to the enamel

### Caries prevalence

**Fig. 2:**
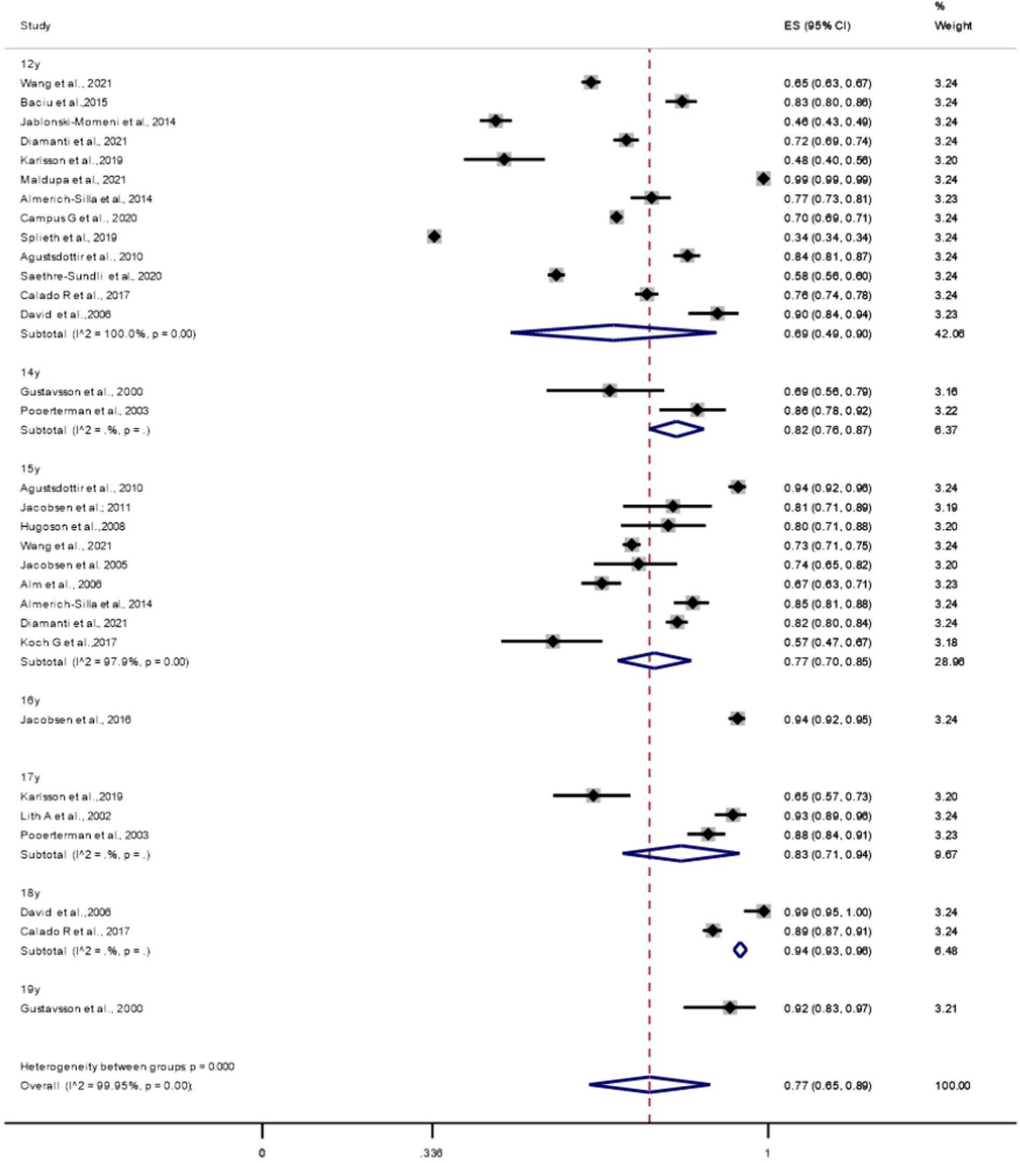
Meta-analysis of caries prevalence when caries is diagnosed at enamel threshold

Of the 25 studies on caries prevalence at the enamel threshold, 22 were included to compute the summary estimates (participants: 84,512; cases with caries: 40,594). As all studies included in the meta-analysis from the Scandinavian countries, diagnosed caries using both clinical- and radiographic examinations, only two non-Scandinavian applied radiographs. Figure 2 shows that the overall prevalence of caries in 12–19-year-old adolescents was 77% (95% CI 49–81%; I^2^ = 99.95%; *P*-_*heterogenity*_ < 0.001). In the subgroup analyses (Table 3), we found a significantly higher caries prevalence among 16–19-year-olds compared with 12–15-year-olds (*P*-_*heterogeneity*_: 0.028). When analyses by age group (12–13 vs. 16–19) were performed, there were still a little evidence of heterogeneity between the groups (*P*-_*heterogeneity*_: 0.057). We also found a significantly higher caries prevalence in adolescents examined before 2010 (1990–2010) than those examined later (*P*-_*heterogeneity*_: 0.001). Especially noticeable among 12-year-olds was a pattern of cross-country variation in caries prevalence. Two studies on German 12-year-olds reported the lowest caries prevalence [45, 46].

**Table 3:** Analyses of caries prevalence, caries experience, and enamel caries as a proportion of total caries experience

Subgroups	*N*	*Summary estimates*	*P*_*heterogenity*_* (within)*	*I*^*2*^	*P*_*heterogenity*_* (between)*
***Caries prevalence (enamel caries threshold)***					
*Age group (years)*					
12–15	24	0.73 (0.59–0.87)	< 0.001	99.9%	0.028*
16–19	7	0.90 (0.85–0.94)	< 0.001	95.2%	
*Publication year*					
< 2010	7	0.83 (0.75–0.89)	< 0.001	96.1%	0.28
≥ 2010	15	0.72 (0.54–0.89)	< 0.001	99.9%	
Examination year*					
< 2010	10	0.78 (0.70–0.87)	< 0.001	98.7%	0.001
≥ 2010	11	0.74 (0.53–0.96)	< 0.001	99.9%	
*Type of examination*					
Partial-mouth	5	0.81 (0.70–0.91)	< 0.001	99.7%	0.48
Full-mouth	17	0.74 (0.57–0.90)	< 0.001	99.9%	
*Europe geographical region*					
Scandinavian	11	0.76 (0.65–0.87)	< 0.001	99.1%	0.88
Non-Scandinavian	11	0.74 (0.53–0.96)	< 0.001	99.9%	
***Caries prevalence (dentine caries threshold)***					
*Publication year*					
< 2010	4	0.56 (0.22–0.90)	< 0.001	99.9%	0.78
≥ 2010	11	0.51 (0.36–0.66)	< 0.001	99.5%	
*Type of examination*					
Partial-mouth	3	0.50 (0.06–0.94	< 0.001	99.9%	0.90
Full-mouth	12	0.53 (0.38–0.68)	< 0.001	99.9%	
*Europe geographical region*					
Scandinavian	9	0.49 (0.32–0.67)	< 0.001	99.9%	0.64
Non-Scandinavian	6	0.57 (0.32–0.81)	< 0.001	99.7%	
***Caries Experience***					
*Age group (years)*					
12–15	17	5.58 (4.33–7.21)	< 0.001	99.7%	0.41
16–19	4	7.61 (3.78–15.30)	< 0.001	98.9%	
*Age grouping (years)*					
*Publication year*					
< 2010	4	5.48 (4.27–8.47)	< 0.001	96.7%	0.74
≥ 2010	10	6.24 (4.16–9.37)	< 0.001	99.9%	
Examination year*					
< 2010	7	7.54 (5.46–10.43)	< 0.001	97%	0.41
≥ 2010	6	5.21 (2.30–11.81)	< 0.001	99%	
*Type of examination*					
Partial-mouth	2	3.53 (1.62–7.70)	< 0.001	93.2%	0.16
Full-mouth	12	6.56 (4.55–9.47)	< 0.001	99.8%	
*Europe geographical region*					
Scandinavian	8	4.43 (2.52–7.81)	< 0.001	98.5%	0.037*
Non-Scandinavian	6	8.89 (6.41–12.33)	< 0.001	99.8%	
***Enamel caries proportion***					
*Age groups (years)*					
12–15	8	0.56 (0.42–0.76)	< 0.001	99.6%	0.10
16–19	3	0.37 (0.24–0.56)	< 0.001	99.8%	

I^2^ = proportion of total variation in effect estimate due to between-study heterogeneity (based on Q)

^*^Karlsson et al. (2019) was excluded because the caries examinations were done both before and after 2010

No indication of publication bias for caries prevalence (LFK index = 0.46; no asymmetry; Additional file 1: 3 and caries experience were apparent (Additional file 1: 4). Further, sensitivity analyses were performed by omitting one study at a time revealed a pooled effect size of dental caries prevalence in the range between 76 to 78% (Additional file 1: 5).

**Fig. 3:**
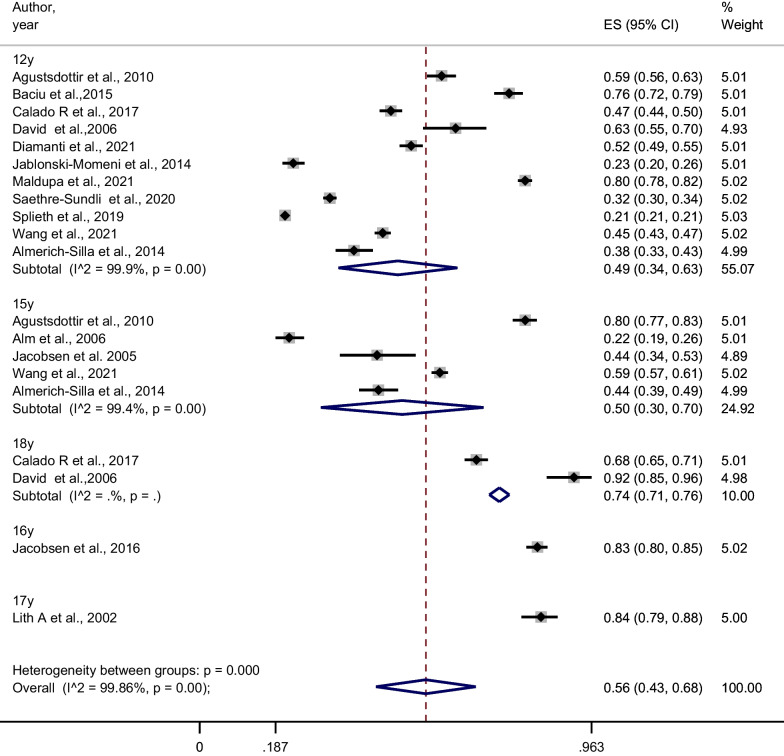
Meta-analysis of caries prevalence when caries is diagnosed at dentine threshold

The overall caries prevalence when performed at the dentine threshold (n = 15 studies), Fig. 3, showed a mean caries prevalence of 56% (95% CI 43–68%; I^2^ = 99.86%; *P*-_*heterogenity*_ < 0.001). Also, caries prevalence at dentine threshold showed no significance difference between studies according to publication year (< 2010 vs. ≥ 2010), mouth examination (full- vs. partial-mouth) and region (Scandinavia vs. rest of Europe) (Table 3). However, the prevalence at dentine level might be overestimated as 5 of the 15 included studies, dentine caries also included the ICDAS Code 3 (visual change in enamel with cavitation).

### Caries experience

**Fig. 4:**
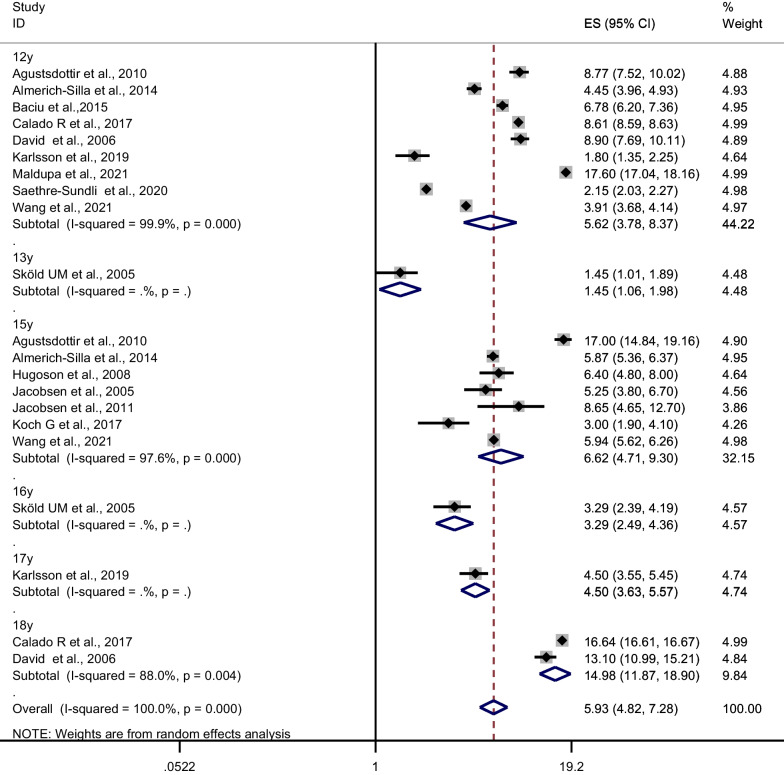
Meta-analysis of caries experience (D[M]FS) when caries is diagnosed at enamel threshold

The present systematic review included 28 publications on caries experience; of these, 14 were eligible for meta-analysis (participants: 17,658). The overall mean estimate of caries experience (DMFS) in 12–19-year-old adolescents was 5.93 (95% CI 4.82, 7.28; I^2^ = 99.9%; P-_heterogenity_ ≤ 0.001) (Fig. 4). Further, the subgroup analyses (Table 2) revealed only significant heterogeneity in caries experience by region with a significantly lower DMFS in Scandinavian countries than in the other European countries in the present study (*P*-_*heterogeneity*_: 0.037). A sensitivity analysis that omitted one study at a time suggested that pooled mean caries experience lies in the range 6.77–7.60 (see Additional file 1: 6).

Further, we found some evidence of publication bias (Egger’s test for a regression intercept; *P* = 0.054), and an asymmetrical funnel plot. However, the evidence of publication bias appears to have been driven by relatively large studies [21, 47–49].We found no evidence of publication bias with Begg’s test (*P* = 0.78).

### The D component—enamel caries

**Fig. 5:**
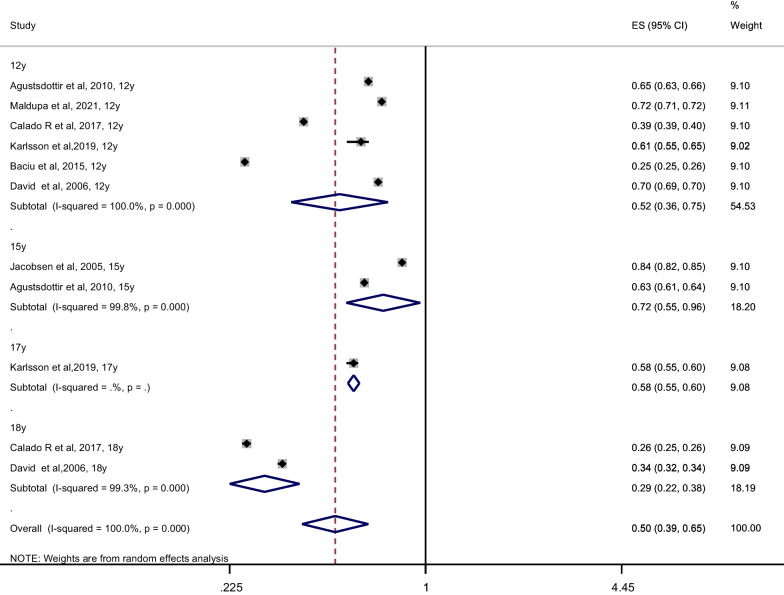
Meta-analysis of proportion of enamel caries

According to Fig. 5, only 7 of the 24 publications were included in meta-analysis computing the summary estimates of enamel caries (participants = 7056). The overall proportion of enamel caries was 0.50 (95% CI 0.39, 0.65; I^2^ = 99.6%; P-_heterogenity_ < 0.001). When we performed a sensitivity analysis deleting one study at a time, the pooled proportion ranged between 0.50–0.57 (see Additional file 1: 7. The proportion of enamel caries in the 12–15-year age group was found to be slightly higher than in the 16–19-year age group, though the *P* for heterogeneity between the groups was non-significant (*P* = 0.10; Table 2). Four of the included 7 studies [48, 50–52] using ICDAS diagnostic criteria, underestimated the proportion of enamel caries because only enamel caries without cavitation was noted, not ICDAS Code 3.

The Swedish studies of 12-and 15-year-olds [53] and of only 15-year-olds [54–56] in the present systematic review, not included in this meta-analysis, found that 80–90% of all proximal caries lesions were enamel caries. Enamel caries as a proportion of total caries (Table 1) was rather low in studies reporting a high total caries experience. As an example, a study from Portugal, published in 2017, found a substantially high caries experience among 12-year-olds (DMFS: 8.6; SD: 0.34) and 18-year-olds (DMFS: 16.64; SD: 0.51) [47] where enamel caries constituted 39% and 26%, respectively, of the total caries burden.

### Caries distribution

No meta-analysis could be conducted since the reporting of caries distribution in the different studies (n = 11 studies) varied too much, both at individual-, tooth- and surface levels.

### At the individual level

Six studies [45, 47–50, 57] used The Significant Caries (SiC) index [58] which measures the mean DMFT for one third of the population with the highest level of caries. The national German study on 12-year-olds [45] found the SiC-index to be three times higher than the mean DMFT of all participating 12-year-olds. Other studies also revealed that caries had a skewed distribution [38, 50, 54, 55]; *e.g.* the 2013 CDHS study in the UK [38] observed that 15% of the 15-year-olds had a severe caries burden. Different measures of socio-economic markers also displayed significant association with caries at individual level (results not shown).

### At the tooth level

In participants aged 12 years, three studies observed the permanent first molars to be the teeth most often affected by caries [21, 48, 59]. One study of these [48] reported the mandibular first molars to be the most caries prone, while another [21] found no difference in caries prevalence between the four quadrants. The same study [21] which also included 15-year-olds, reported that the permanent second molars at that age were increasingly more caries prone. The teeth least affected by caries among 12- and 15-year-olds, were the lower anterior- and upper canine teeth [21]. By age 18 years, the first permanent molars had still the highest caries experience [59].

### At the surface level

Some publications reported that the caries surfaces most often affected among 12- and 15-year-olds were the occlusal surfaces of the permanent molars and the buccal surfaces of the lower first molars [21, 46, 59]. In Sweden, however, the Jönköping epidemiological surveys in 15-year-olds [54, 55], reported that proximal surfaces were most often affected with caries of all surfaces. The 2013 CDHS study targeting 15-year-olds also revealed that the surface distribution of caries was influenced by the extent of the caries experience [37]; among those with low decay caries experience, caries mainly affected the occlusal and buccal surfaces of the permanent molars, but among those with extremely high decay experience, caries lesions affected almost all teeth, even the anterior surfaces of mandibular teeth.

## Discussion

This systematic review and meta-analysis report on dental caries also including enamel caries among European adolescents. First, the studies included had a substantial level of statistical variability. The meta-analyses of caries prevalence suggested that 77% of the adolescents were affected by caries (n = 22 studies), with a significantly higher caries prevalence in 16–19-year-old group. Caries prevalence was also significantly higher among participants examined before 2010 compared with in 2010 and after, which indicates a caries reduction in recent years. Our meta-analysis of caries experience (n = 14) found significantly lower value among adolescents in Scandinavian countries than in European countries outside Scandinavia. In the meta-analysis of enamel caries proportion, it constituted 50% of the total caries experience (n = 7 studies); however, this proportion was higher in the 12–15 year than the 16–19-year age group. Other publications that were not included in the meta-analysis tended to confirm this finding, reporting enamel caries to constitute 80–90%. Thus, our findings have clearly revealed that when caries epidemiology omits consideration of enamel caries, the caries burden is seriously underreported. The systematic review also contained information about the distribution of caries (n = 11 studies). This information also confirmed findings in the literature that caries distribution was skewed, both at individual-, tooth- and surface level. At tooth and surface level, this distribution also changed according to age.

The present findings were not representative of the European continent since the search resulted in studies originated in only one-fourth of the countries and only a share of these reported caries on national levels [21, 37, 45, 47, 48, 50, 57, 60]. Germany reported the lowest caries prevalence with data for 12-year-olds [45, 46], but since bitewing radiographs were not taken, caries prevalence may be underestimated [46, 50]. The lower caries prevalence in Germany and sometimes in Scandinavia, may be due to the organization of dental health care and the focus on preventive care for this age group; free dental health care service in Germany through a comprehensive oral health insurance [61] and in Scandinavia, through publicly free provided oral healthcare services [62]. Although many countries in Southern Europe provide free public dental services for children, dental treatment of adolescents may still incur out-of-pocket costs [63]. Caries distributions at the individual level (not shown by meta-analysis) have indicated that a multitude of socio-demographic markers of caries also prevail in countries with free dental health care. As European countries are not homogeneous, a validated, measure comprising socio-economic markers would have benefitted our review by allowing inter-country comparisons [64].

It has been reported that enamel caries has a greater impact on caries estimates among school children with a higher SES compared with among those with a lower SES [65] and that enamel caries is more often a higher proportion of total caries in populations with low compared with high caries prevalence [66]. The dominance of enamel caries seen in 12- and 15-year-olds in Scandinavia (countries with a high HDI) [53–56] is consistent with this literature. Because caries progression is lower in individuals living in affluent conditions, the reasoning is that enamel caries is more likely to be identified [65].

Current knowledge that caries increases with age is consistent with this present meta-analysis of caries prevalence, showing a significantly higher prevalence in the 16–19-year-old group. We also observed higher DMFS scores in the older age groups compared with younger age groups, but the differences were not significant. The lack of significance may be both methodological and biological: methodologically, due to the high degree of clinical heterogeneity (*e.g.,* inconsistency in sample size) [67] and biologically, due to the variability of caries risk during the adolescent years. The occlusal surfaces of the permanent second molars are at highest caries risk the first 3 years after eruption, during ages 12–15 years [12]. Likewise, following eruption and establishment of proximal contact in this same period, proximal surfaces of premolars and molars are at likelihood of new caries lesions [12], in particular the distal surfaces of the premolars and the mesial surfaces of the second molars [13]. Lesion progression from the enamel into the dentine, however, is reported to be relatively slow; surfaces affected by enamel caries survive a median of 4.8 years and 46% of enamel caries survive 15 years without progressing into dentine [12, 13]. This implies that enamel lesions most often occur in early adolescence and then progress during late adolescence. Mejàre and Kidd [68] observed that a caries-free 15–16-year-old runs a very small risk of experiencing new lesions over the next 3 years. It is therefore essential that especially during early adolescence, the great prevention potential visualized by the volume of enamel caries, should be fully exploited. When studies omit consideration of enamel lesions, the caries data simply demonstrate a failure of the optimal treatment option: the one being performed when the lesions were in the enamel stage.

The meta-analyses of both caries prevalence and overall caries experience did not differ significantly between partial- vs. full mouth examination. This finding is in line with the Swedish Jönköping surveys in 15-year-olds [54, 55], which found consolidation of proximal caries to be extensive during adolescence. The 2013 CDHS study from the UK showed that the level of caries experience influenced caries distribution [37]: the distribution of caries lesions among participating 15-year-olds differed between groups with low and extremely high decay experience. This supports the model of Batchelor and Sheiham, introduced 20 years ago, of grouping tooth surfaces by caries susceptibility [69].

### Strengths

The most important strength of the present systematic review and meta-analysis was the inclusion of enamel caries in the definition of caries burden during the searches, thus allowing both the magnitude of enamel caries and its proportion of the total caries experience to be quantified. Including enamel lesions in the selection criteria means the present systematic review and meta-analysis is the first to accurately reflect modern dental caries epidemiology [70]. Our systematic review also looked at the distribution of lesions at the individual-, tooth-, and surface- levels, issues that were emphasized in the 2018 “Brussels statement on the future needs for caries epidemiology and surveillance in Europe”[64].

### Limitations

Only limited studies could be included in the meta-analysis on the enamel proportion because most of the studies have not reported standard deviation or confidence intervals. Its meta-analysis result was also underestimated because in four out of seven included studies, accurate estimations of enamel caries were not possible. Other shortcomings were that some of the included publications provided little information on previous calibration procedures, some publications did not report the number of examiners or reported a high number, and use of bitewing radiography varied. Together with the skewed distribution of ages and fewer studies fulfilling the inclusion criteria outside Scandinavia, the present findings might not be considered representative of the European adolescent population.

## Conclusion

Although studies in which the caries examinations had been done in 2010 or later documented a reduction in caries prevalence, caries during adolescence still constitutes a burden. Thus, the potential for preventing development of more severe caries lesions, as seen in the substantial volume of enamel caries during early adolescence, should be fully exploited. For this to happen, enamel caries should be a part of epidemiological reporting in national registers.

## Supplementary Information

**Additional file 1**. Seach strategy in four electronic databases; Medline Ovid, Embase, CINAHL, Sewed+ (Sept 20th 2021).

## Data Availability

All data generated and analyzed during this study are included in this published article [and its supplementary information files].

## Abbreviations

BW: Bitewing radiographs
CoCoPop: Condition, Context, and Population
DMFT/DMFS: Decayed/missed/filled permanent teeth/surfaces
FAS: Family Affluence Scale
ICDAS: International Caries Detection and Assessment System
IMD: Indices of Multiple Deprivation
HDI: Human Development Index
JBI: Joanna Brigg’s Institute
RCTs: Randomized Controlled Trials
WHO: World Health Organisation
