# Correction: Dental caries at enamel and dentine level among European adolescents – a systematic review and meta-analysis

**DOI:** 10.1186/s12903-023-02819-0

**Published:** 2023-02-19

**Authors:** Marit S. Skeie, Abhijit Sen, Göran Dahllöf, Tone Natland Fagerhaug, Hedda Høvik, Kristin S. Klock

**Affiliations:** 1 Department of Clinical Dentistry, The Faculty of Medicine, Pediatric Dentistry, University of Bergen, Årstadveien 19, 5009 Bergen, Norway; 2 Center for, Oral Health Services and Research, Mid-Norway (TkMidt), Trondheim, Norway; 3 Department of Public Health and Nursing, Faculty of Medical and Health Sciences, Norwegian University of Science and Technology (NTNU), Trondheim, Norway; 4 Division of Orthodontics and Pediatric Dentistry, Department of Dental Medicine, Karolinska Institutet, Stockholm, Sweden

**Correction: BMC Oral Health (2022) 22:620** 10.1186/s12903-022-02631-2

In this article, there is a typo in the result section of the Abstract.

**Original Abstract text**: “The overall mean DMFT score was 5.93 (n = 14 studies) and……”

**Revised Abstract text**: “The overall mean DMFS score was 5.93 (n = 14 studies) and……”

The original article has been corrected.

