# Supplementary material for: Dental caries at enamel and dentine level among European adolescents – a systematic review and meta-analysis
Source: BMC Oral Health. 2022 Dec 18;22:620. doi: 10.1186/s12903-022-02631-2 (PMC9762106; doi:10.1186/s12903-022-02631-2)
Supplement: Supplementary file 1 — Additional file 1. Seach strategy in four electronic databases; Medline Ovid, Embase, CINAHL, Sewed+ (Sept 20th 2021). [file 12903_2022_2631_MOESM1_ESM.docx]

**Supplementary Material**

**Additional file 1. Seach strategy in four electronic databases; Medline Ovid, Embase, CINAHL, Sewed+ (Sept 20^th^ 2021).**

**Search strategy in Medline Ovid**

|  |  |  |
| --- | --- | --- |
| 1 | Caries.ti,ab,kw. | (45201) |
| 2 | exp Dental Caries/ | (47902) |
| 3 | 1 or 2 | (62139) |
| 4 | (Enamel caries or Initial caries or Enamel defect* or Enamel cavity or Enamel cavities or Caries Treshold* or Dentine caries or Manifest caries or Enamel or Dentin or Dentine or ICDAS or "International Caries Detection and Assessment System" or level*).ti,ab,kw. | (4358291) |
| 5 | (Pattern or Prevalence or Epidemiology or Incidence or occurrence).ti,ab,kw | (2551289) |
| 6 | exp Prevalence/ | (315987) |
| 7 | exp Epidemiology/ | (27672) |
| 8 | exp Incidence/ | (281442) |
| 9 | 5 or 6 or 7 or 8 | (2718357) |
| 10 | (Adolescen* or Teenage* or Young people or students or school children or permanent teeth or permanent tooth).ti,ab. | (605670) |
| 11 | exp Adolescent/ | (2121623) |
| 12 | exp Child/ | (2005315) |
| 13 | 10 or 11 or 12 | (3439381) |
| 14 | 3 and 4 and 9 and 13 | (2805) |
| 15 | limit 14 to yr="2000 - 2021" | (2061) |

**Search strategy in Embase**

|  |  |  |
| --- | --- | --- |
| 1 | Caries.ti,ab,kw | (42334) |
| 2 | exp Dental Caries/ | (50382) |
| 3 | 1 or 2 | (60199) |
| 4 | (Enamel caries or Initial caries or Enamel defect* or Enamel cavity or Enamel cavities or Caries Treshold* or Dentine caries or Manifest caries or Enamel or Dentin or Dentine or ICDAS or "International Caries Detection and Assessment System" or level).ti,ab,kw. | (2881409) |
| 5 | (Pattern or Prevalence or Epidemiology or Incidence or occurrence).ti,ab,kw. | (3414013) |
| 6 | exp Epidemiology/ | (3807479) |
| 7 | 5 or 6 | 5811554) |
| 8 | (Adolescen* or Teenage* or Young people or students or school children or permanent teeth or permanent tooth).ti,ab. | (778014) |
| 9 | exp Adolescent/ | (1613165) |
| 10 | exp Child/ | (2780730) |
| 11 | 8 or 9 or 10 | (3915582) |
| 12 | 3 and 4 and 7 and 11 | (3174) |
| 13 | Limit 12 to vr=”2000 – 2021” | (2492) |

**Search strategy in CINAHL**

| **#** | **Query** | **Results** |
| --- | --- | --- |
| S1 | MH Dental caries | 12,976 |
| S2 | TI caries | 5,957 |
| S3 | AB caries | 9,136 |
| S4 | TI Enamel caries OR Initial caries OR Enamel defect* OR Enamel cavity OR Enamel cavities OR Caries Treshold* OR Dentine caries OR Manifest caries OR Enamel OR Dentin OR Dentine OR ICDAS OR “international Caries Detection and Assessment System” | 4,014 |
| S5 | AB Enamel caries OR Initial caries OR Enamel defect* OR Enamel cavity OR Enamel cavities OR Caries Treshold* OR Dentine caries OR Manifest caries OR Enamel OR Dentin OR Dentine OR ICDAS OR “international Caries Detection and Assessment System” | 6,900 |
| S6 | MH child | 485,069 |
| S7 | MH adolescence | 556,140 |
| S8 | TI Adolescen* OR Teenage* OR Young people OR students OR school children OR permanent teeth OR permanent tooth | 203,814 |
| S9 | AB Adolescen* OR Teenage* OR Young people OR students OR school children OR permanent teeth OR permanent tooth | 282,984 |
| S10 | MH Epidemiology | 5,925 |
| S11 | TI Pattern* OR Prevalence OR Epidemiolog* OR Incidence | 140,455 |
| S12 | AB Pattern* OR Prevalence OR Epidemiolog* OR Incidence | 523,015 |
| S13 | S1 OR S2 OR S3 | 16,424 |
| S14 | S4 OR S5 | 8,267 |
| S15 | S6 OR S7 OR S8 OR S9 | 1,004,121 |
| S16 | S10 OR S11 OR S12 | 585,655 |
| S17 | S13 AND S14 AND S15 AND S16 | 263 |
| S18 | S13 AND S14 AND S15 AND S16 | 257 |

**Search strategy in Svemed+ (available up to 2020)**

| **Nr** | **Söksträng** | **Antal träffar** |
| --- | --- | --- |
| 8 | exp:"Dental Caries" | 520 |
| 12 | exp:"Adolescent" | 9922 |
| 13 | exp:"child" | 11791 |
| 14 | exp:"prevalence" | 1123 |
| 15 | exp:"epidemiology" | 182 |
| 16 | exp:"incidence" | 1054 |
| 17 | 12 OR 13 | 16774 |
| 18 | 14 OR 15 OR 16 | 2200 |
| 19 | 8 AND 17 AND 18 | 24 |

**Additional file 2**

Critical quality appraisal (The Joanna Briggs Institute Appraisal Instrument for Studies Reporting Prevalence Data was used, but scoring modified in support of this systematic review and multi-analysis). The first part of the table consists of publications based on full mouth caries examination and the second one, on publications from partial caries examination (proximal surfaces of posterior teeth). Within these sections, the publications are presented consecutively according to publication date.

|  | **Q1** | **Q 2** | **Q 3** | **Q 4** | **Q 5** | **Q 6** | **Q 7** | **Q 8** | **Q 9** | **Total sum** | |
| --- | --- | --- | --- | --- | --- | --- | --- | --- | --- | --- | --- |
| ***Full mouth caries examination*** | | | | | | | | | | |  |
| Saethre-Sundli HB et al. 2020 | * | * | * | ** | * | ** | * | * | * | 11 | |
| Jacobsen ID et al. 2016 |  | * | * | ** | * | ** | * | * | * | 10 | |
| David J et al. 2006 | * |  |  | ** | * | ** | * | * | * | 9 | |
| Karlsson F et al. 2019 | * | * | * | ** | * | * | * | * | * | 10 | |
| Koch G et al. 2017 | * | * |  | ** | * | ** | * | *? | *? | 10 | |
| Jacobsen B et al. 2011 | * | * |  | ** | * | ** | * | * | * | 10 | |
| Hugoson A al. 2008 | * | * |  | ** | * | ** | * | * |  | 9 | |
| Agustsdottir H et al. 2010 | * | * | * | * | * | ** | * | * | * | 10 | |
| Splieth CH et al. 2010 | * | * | * | * | * | * | * | * | * | 9 | |
| Jablonski-Momeni A et al. 2014 | * | * | * | * | * | * | * | * | * | 9 | |
| The 2013 Children’s Dental Health Survey (CDHS) data from UK were represented by 3 publications | | | | | | | | | | |  |
| Wang X et al. 2021, Wang X et al. 2021, Vernazza CR et al. 2016 | * | * | * | ** | * | * | * | * | * | 10 | |
| Baciu D et al. 2015 | * | * | * | ** | * | * | * | * | * | 10 | |
| Maldupa I et al. 2021 | * | * | * | ** | * | * | * | * | * | 10 | |
| Deery C et al. 2000 | * | * |  | ** | * | ** | * | * | * | 10 | |
| The 2010 survey from the Valencia region of Spain was represented by 3 publications | | | | | | | | | | |  |
| Almerich-Torres T et al. 2020, Almerich-Torres T et al. 2017, Almerich-Silla JM et al. 2014 | * | * | * | ** | * | * | * | * |  | 9 | |
| Calado R et al. 2017 | * | * | * | ** | * | * | * | * |  | 9 | |
| Campus G et al. 2020 | * | * | * | ** | * | * | * | * | * | 10 | |
| Diamanti I et al. 2021 | * | * | * | ** | * | * | * | * |  | 9 | |
| ***Partial mouth caries examination (approximal surfaces of posterior teeth). One study also included occlusal surfaces.*** | | | | | | | | | | |  |
| Jacobsen ID et al. 2019 | Data based on Jacobsen ID et al. 2016 referred above. Two publications derived from “Fit Futures” study in Troms county, Norway. | | | | | | | | | |  |
| Bergström EK et al. 2019 | * | * | * | ** | * | ** | * | * | * | 11 | |
| Alm A et al. 2006 | * | * | * | ** | * | ** | * | * | * | 11 | |
| Sköld UM et al. 2005 | * | * | * | ** | * | ** | * | * | * | 11 | |
| Jacobsson B et al. 2005 | * | * | * | * | * | * | * | * | * | 9 | |
| Lith A et al. 2002 | * | * | * | ** | * | ** | * | * | * | 11 | |
| Gustavsson A et al. 2000 | * | * |  | ** | * | ** |  | * |  | 8 | |
| Pooerterman JHG et al. 2003 | * |  |  | * | * | ** | * | * | * | 8 | |

*The following questions (Qs) were: Q1. Was the sample frame appropriate to address the target population? Q2. Were study participants sampled in an appropriate way? Q3. Was the sample size adequate? Q4. Were the study subjects and the setting described in detail? Q5. Was the data analysis conducted with sufficient coverage of the identified sample? Q6. Were valid methods used for the identification of the condition? Q7. Was the condition measured in a standard, reliable way for all participants? Q8. Was there appropriate statistical analysis? Q9. Was the response rate adequate, and if not, was the low response rate managed appropriately? Concerning Q4 and Q6, a maximum of two stars (two points) could be awarded. All other questions had one star (point) as maximum, making the range of scores to be from 0 to 11.

**Additional file 3.** DOI plot for assessing publication bias for caries prevalence


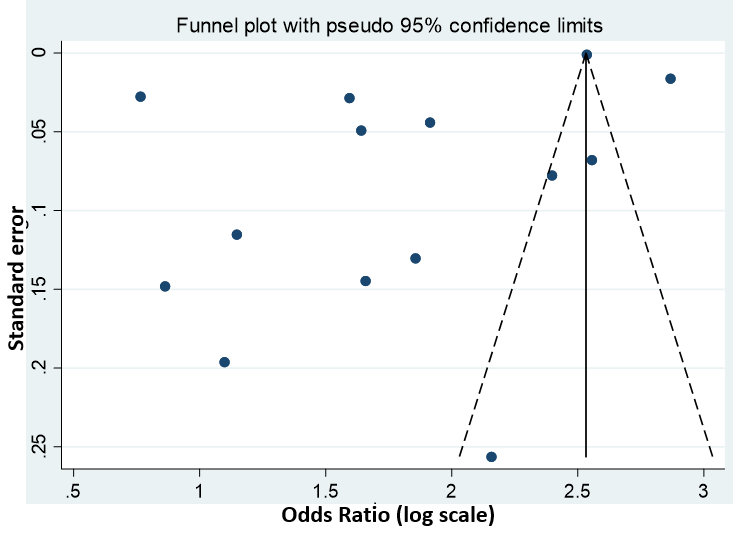


**Additional file 4**. Funnel plot for assessing publication bias of caries experience.

**Additional file 5.** Influence analyses of caries prevalence by omitting one study at a time).

**Additional file 6.** Influence analyses of caries experience (by omitting one study at a time).

**Additional file 7.** Influence analyses of enamel caries proportion (by omitting one study at a time).
